# WITCH-NG: efficient and accurate alignment of datasets with sequence length heterogeneity

**DOI:** 10.1093/bioadv/vbad024

**Published:** 2023-03-06

**Authors:** Baqiao Liu, Tandy Warnow

**Affiliations:** Department of Computer Science, University of Illinois Urbana-Champaign, Champaign, IL 61820, USA; Department of Computer Science, University of Illinois Urbana-Champaign, Champaign, IL 61820, USA

## Abstract

**Summary:**

Multiple sequence alignment is a basic part of many bioinformatics pipelines, including in phylogeny estimation, prediction of structure for both RNAs and proteins, and metagenomic sequence analysis. Yet many sequence datasets exhibit substantial sequence length heterogeneity, both because of large insertions and deletions in the evolutionary history of the sequences and the inclusion of unassembled reads or incompletely assembled sequences in the input. A few methods have been developed that can be highly accurate in aligning datasets with sequence length heterogeneity, with UPP one of the first methods to achieve good accuracy, and WITCH a recent improvement on UPP for accuracy. In this article, we show how we can speed up WITCH. Our improvement includes replacing a critical step in WITCH (currently performed using a heuristic search) by a polynomial time exact algorithm using Smith–Waterman. Our new method, WITCH-NG (i.e. ‘next generation WITCH’) achieves the same accuracy but is substantially faster. WITCH-NG is available at https://github.com/RuneBlaze/WITCH-NG.

**Availability and implementation:**

The datasets used in this study are from prior publications and are freely available in public repositories, as indicated in the Supplementary Materials.

**Supplementary information:**

Supplementary data are available at *Bioinformatics Advances* online.

## 1 Introduction

Multiple sequence alignment (MSA) is a fundamental task in computational biology and is a prerequisite for many downstream analyses such as phylogeny estimation (Morrison, 2006), metagenomics (Matsen *et al.*, 2010) and other applications. Over recent years, the assembly of large sequence datasets has led to the development of MSA methods that are able to scale to very large datasets [e.g. Clustal-Omega (Sievers *et al.*, 2011)] as well as techniques that use divide-and-conquer to maintain high accuracy [e.g. PASTA (Mirarab *et al.*, 2015) and MAGUS (Smirnov and Warnow, 2021a)]. Yet, accurate MSA estimation still remains challenging, especially under conditions such as high rates of evolution or sequence length heterogeneity.

**Fig. 1.**
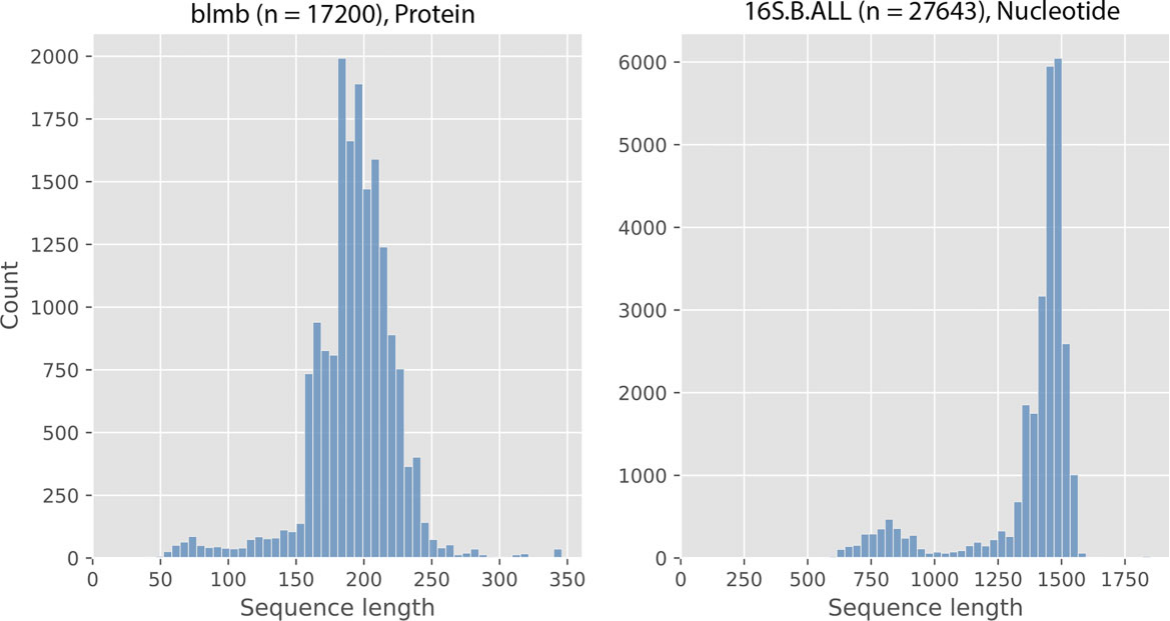
Sequence length histograms of two biological datasets that show sequence length heterogeneity. ‘blmb’ comes from HomFam (Sievers *et al.*, 2011) and ‘16S.B.ALL’ comes from the Comparative RNA Website (CRW) (Cannone *et al.*, 2002). *n* denotes the number of sequences in the dataset

Sequence length heterogeneity, in particular the presence of many short sequences, is a frequent characteristic of biological datasets (Fig. 1 contains two examples). Sequence length heterogeneity can arise due to various reasons, such as the inclusion of many short reads or partially assembled sequences, or purely from evolutionary events such as domain-level deletions. MSA methods that are accurate on datasets without sequence length heterogeneity can degrade severely in accuracy under substantial presence of fragments, and the resulting alignments will in-turn adversely affect downstream analyses (Smirnov and Warnow, 2021b). Therefore, specialized MSA estimation methods that are robust to sequence length heterogeneity are valuable.

One effective approach for aligning datasets with sequence length heterogeneity selects a set of ‘full-length’ sequences from the input, aligns these sequences and then adds the rest of the sequences (the ‘queries’) into the computed ‘backbone’ alignment. The last step where sequences are added into an existing alignment is a necessary step in other applications, including updating alignments, updating phylogenies (i.e. phylogenetic placement) (Czech *et al.*, 2022), remote homology detection (Nguyen *et al.*, 2016) and taxon identification and abundance classification in metagenomics (Mirarab *et al.*, 2011). Methods that can add sequences into existing alignments are available in HMMER (Eddy, 2011; Finn *et al.*, 2011) (using the popular hmmbuild+hmmalign pipeline), PaPaRa (Berger and Stamatakis, 2011), SEPP (Mirarab *et al.*, 2011), MAFFT (Katoh and Standley, 2013) (using the—add option), UPP (Nguyen *et al.*, 2015) and WITCH (Shen *et al.*, 2022b).

WITCH is the most recent of these methods for adding sequences into an existing alignment. WITCH uses a two-stage technique to add sequences into a backbone alignment. In the first stage, it computes an ensemble of Hidden Markov Models (eHMM) to represent the backbone alignment, using tools from HMMER. In the second stage, WITCH adds the remaining sequences (i.e. the query sequences) into the backbone alignment, using the eHMM. At a high level, this two-stage structure is the same as in UPP, but WITCH executes the second stage differently. While UPP simply picks a single HMM from the eHMM to add a given query sequence into the backbone alignment, WITCH computes an extended alignment for the query sequence for each of the HMMs in the ensemble, and then combines the top (default 10) of these extended alignments, weighted by the probabilities it associates to each HMM in the ensemble, into a single extended alignment using the ‘Graph Clustering Merger’ (GCM) from MAGUS (Smirnov and Warnow, 2021a).

The resulting pipeline improves the accuracy of UPP when aligning datasets that have evolved under a high rate of evolution and otherwise matches UPP, but is somewhat slower (Shen *et al.*, 2022b). Since WITCH and UPP differ only in the last step, this shows that the runtime cost is a result of its weighted consensus step where WITCH uses the top 10 HMMs in the eHMM instead of just one, and more importantly because WITCH uses GCM, a general alignment merging method, for merging all the information in the different alignments (one for each HMM) in order to add the query sequence into the backbone alignment.

Here, we present WITCH-NG, a fast way of implementing the same algorithmic strategy as WITCH. Most importantly, we replace the computationally intensive and complicated GCM technique by a simple use of the polynomial time local alignment method, Smith–Waterman (Smith and Waterman, 1981), to add each query sequence into the backbone alignment, and this change achieves the same accuracy at a fraction of the runtime. Moreover, this innovation provides a new way of solving this basic bioinformatics problem, and so has broad applicability. WITCH-NG by design matches the accuracy of WITCH but is much faster (and nearly as fast as UPP). Like WITCH, WITCH-NG is more accurate than UPP.

## 2 Background

### 2.1 WITCH

WITCH has five basic phases. In Phase 0, the input sequences are divided into a set of backbone sequences and the remaining sequences (called ‘query sequences’), with the backbone sequences sampled from those sequences deemed to be full-length. An alignment (default: MAGUS) and its maximum likelihood tree [default: FastTree2 (Price *et al.*, 2010)] are then constructed for the backbone sequences. In Phase 1, an eHMMs is built on the backbone alignment. Phases 2 and 3 are then used to define an ‘extended alignment’ for each query sequence, which is an alignment that contains the backbone alignment and the given query sequence. Phase 4 then combines the information in different extended alignments, one per query sequence, using transitivity, so that the output is an alignment of the entire set of sequences that induces the constructed extended alignments.

Since Phases 2 and 3 together are the most complex and together define how to add a given query sequence into the backbone alignment, we describe them in detail here. For a given query sequence, Phase 2 operates by computing a weighted score (larger is better) for each HMM in the ensemble, and then picking the top *k* such HMMs (default: k=10). Phase 3 has several steps that we now describe, which jointly constitute a weighted version of the Graph Clustering Merger (GCM) from MAGUS. In Phase 3(a), it builds an extended alignment for the query sequence for each of these *k* selected HMMs and uses these to construct an ‘alignment graph’ where the nodes of the graph correspond to the positions in the query sequence or the backbone alignment and the edges are present between two nodes if and only if the corresponding positions are aligned in one of these extended alignments. These edges are weighted by the support defined by these extended alignments. In Phase 3(b), this weighted alignment graph is clustered using Markov Clustering (MCL), described initially in Dongen (2000), which partitions the nodes into disjoint clusters. Since this set of clusters may not be a ‘valid trace’ (Kececioglu, 1993) (i.e. it may not define a valid pairwise extended alignment of the query sequence to the backbone alignment), Phase 3(c) then runs an A* algorithm to post-process the clustering to make it consistent with a valid alignment. Thus, the output of Phases 2 and 3 is an extended alignment for each query sequence (i.e. an alignment containing the query sequence and the backbone alignment).

## 3 WITCH-NG: redesign of WITCH

**Fig. 2.**
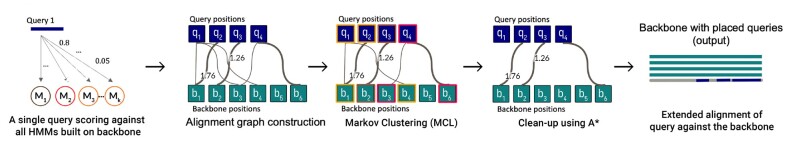
Overview of Phases 2 and 3 of the original WITCH algorithm (applied to a single query sequence). Given a single query sequence and the ensemble of HMMs, in Phase 2 a fitness score of that query is derived for each HMM in the ensemble. In Phase 3(a), for each of the top k=10 HMMs, an alignment of the query to the backbone alignment (i.e. an ‘extended alignment’) is constructed, and the pairwise homologies define a bipartite ‘alignment graph’ with weights on the edges indicating the support for that homology (see text for more details). Phase 3(b): WITCH then runs Markov clustering (MCL) on the weighted alignment graph, producing a clustering of the nodes. If the modified graph may contain ‘violations’—i.e. sets of homologies (edges) that cannot coexist in an alignment—then a post-clustering Phase 3(c) occurs. In this post-clustering analysis, WITCH runs an A* heuristic search to modify the clustering to remove violations, so that it defines a ‘valid trace’ (i.e. a valid alignment). The MCL and A* methods are the computationally intensive parts of Phase 3 in the WITCH pipeline. This figure is partially based on Figure 2 from Nguyen *et al.* (2015) used under the CC BY 4.0 license

The key difference between WITCH and WITCH-NG is how each query sequence *q* is added to the backbone alignment. In WITCH, this is accomplished during Phases 2 and 3, where for each query sequence a weighted alignment graph is computed, then passed to MCL, then passed to an A* method (to remove violations) so that the final output is a valid extended alignment for each query sequence (see Fig. 2 for how WITCH handles Phase 3). MCL can be computationally intensive and employs randomness to perform the graph clustering, and the A* algorithm is also often computationally intensive. Thus, while Phases 2 and 3(a) are both fast and deterministic, Phases 3(b) (running MCL) and 3(c) (running the A* method) are not fast and due to reliance on randomness, are not even predictable in accuracy or runtime.

**Fig. 3.**
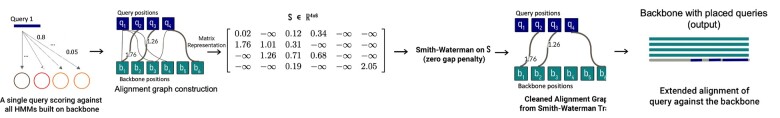
WITCH-NG replaces Phases 3(b) and 3(c) in the WITCH pipeline. The information in the alignment graph (Step 3(a) in the WITCH pipeline) is used to define the scoring matrix *S*, where *S[x*, *y]* is the reward in matching position *x* in the query sequence to position *y* in the backbone alignment (and with S[x,y]=−∞ if the two positions are not aligned by one of the extended alignments used to construct the alignment graph). This matrix is then used by Smith–Waterman, with zero cost gap penalty, to produce the alignment of the query sequence to the backbone alignment. The final output from WITCH-NG may be the same as for WITCH since they both effectively try to solve the same optimization problem (one explicitly and the other implicitly), but WITCH-NG solves the problem exactly in polynomial time while WITCH uses computationally intensive heuristics. This figure is partially based on Figure 2 from Nguyen *et al.* (2015) used under the CC BY 4.0 license

In WITCH-NG, the calculation of the extended alignment for each query sequence is handled differently. Phase 2 (scoring the query sequence against each HMM in the ensemble and computing the weight) and Phase 3a (keeping the top k=10 HMMs and using them to construct the weighted alignment graph) are the same. However, WITCH-NG replaces Phases 3b and 3c (compare Figs 2 and 3), as we now describe. Phases 3(b) and 3(c), which run MCL and then the A* method to produce a valid trace, are replaced by a much simpler approach: the weighted alignment graph is used to define a scoring matrix *S*, where *S*[*x*, *y*] denotes the reward of matching position *x* in the query sequence to position *y* in the backbone sequence, and where S[x,y]=−∞ if *x* and *y* are not aligned by any of the top *k* HMMs (to forbid aligning two positions that are not supported by any of the selected HMMs). The local alignment method, Smith–Waterman (Smith and Waterman, 1981), is then run using *S* as a scoring matrix with zero cost gap penalty, to align the query sequence to the backbone alignment. As a result of this simplification, WITCH-NG achieves the same alignment accuracy as WITCH, but in a fraction of the time.

We now provide some intuition as to why the use of local alignment (computed using Smith–Waterman) instead of the Graph Clustering Method (GCM) produces alignments of very similar accuracy. Zaharias *et al.* (2022) have argued that there is an implicit optimization problem in GCM that is identical to the solution to the local alignment problem using this cost function. Specifically, Zaharias *et al.* showed that GCM is a good heuristic for the optimization problem ‘Maximum Weight Trace for Alignment Merging (MWT-AM)’ [a generalization of the classical Maximum Weight Trace (MWT) problem (Kececioglu, 1993) in bioinformatics]. In short, MWT-AM defines an optimization criterion to the scenario when merging multiple disjoint ‘constraint’ alignments (i.e. the homologies in these alignments cannot be altered in the merging process) and when similarity scores between the constraint alignment columns have been obtained. Moreover, Zaharias *et al.* (2022) also showed that maximizing the MWT-AM score for merging many alignments is beneficial for alignment accuracy.

Given that the original WITCH always uses GCM to merge two alignments, we consider the problem of optimizing for the MWT-AM criterion (restricted to the case of two alignments). We give the definition of MWT-AM on two alignments below:

Definition 1 (MWT-AM, trivial case) Given a weighted undirected bipartite graph with nodes $q_{1},\ldots ,q_{m}$ and $b_{1},\ldots ,b_{n}$, edges of form $\left(q_{i},b_{j}\right)$ with weight function $w((q_{i},b_{j}))>0$, select a subset of edges T (the ‘trace’) maximizing

subject to an additional ‘non-crossing’ constraint, where for any two different $\left(q_{i},b_{j}\right)$ and $\left(q_{x},b_{y}\right)$ in *T*, either both i<x and j<y, or both x<i and y<j.

$$\sum_{(q_{i},b_{j})\in T}w((q_{i},b_{j}))$$

This trivial case is solvable in O(mn) time and O(mn) space by a simple DP algorithm [i.e. a simplified version of either Smith–Waterman or Needleman and Wunsch (1970) without gap penalty], which coincides with our presented algorithm on *S*. This observation is far from new and is mentioned in the paper introducing the maximum weight trace (MWT) problem (Kececioglu, 1993).

Therefore, WITCH-NG can be seen as replacing a computationally intensive heuristic (i.e. GCM) for the MWT-AM problem by an exact polynomial time algorithm for the same problem.

### 3.1 Implementation

We note two major differences in the implementation of WITCH-NG aiming for better running time compared to WITCH. Our first change is a strict improvement to the very last step of WITCH before the output, when the extended alignments (alignments only containing a query sequence and the backbone) are transitively merged. The original implementation in WITCH not only conceptually builds these extended alignments but also writes these extended alignments to the disk verbatim before consuming them using a generic transitivity merging subroutine. This strategy is heavy in IO usage (writing extended alignments to disk then parsing them back into memory) and is extraneous. In WITCH-NG, we directly memorize each query letter’s matched position in the backbone, which is sufficient to output the final alignment without incurring extra runtime cost in IO or parsing.

The second difference is that WITCH-NG implements a different strategy in invoking hmmsearch (the bottleneck step in obtaining the bitscores for UPP and in turn the weights for WITCH). WITCH-NG aggressively avoids invoking extra IO, and especially disk writes. Both WITCH and UPP use temporary files to divide up the query sequences into chunks for hmmsearch to consume and also to save outputs of hmmsearch across often times hundreds of HMMs. Combined with the IO usage of hmmsearch reading the HMMs (saved on disk), these steps might hinder efficient parallelization. In other words, operations can become IO-bound instead of CPU-bound, in which case adding more cores will not speed up the computation of this bottleneck. To allow efficient parallelization under many cores, WITCH-NG instead relies on piping small chunks of sequences to the hmmsearch command while also parsing the hmmsearch output in-memory to minimize IO operations. This simple design allows more cores to be efficiently used until the IO bottleneck is hit.

## 4 Experimental design

We assembled a diverse set of publicly available datasets (both simulated and biological, locations provided in Supplementary Material Section S3), to evaluate the difference of WITCH-NG and WITCH, along with WITCH’s predecessor UPP. The list of datasets and their respective statistics are shown in Table 1, where simulated conditions have been intentionally fragmented [1000M-HF series have half of the sequences fragmented to roughly 250 bp in length (Smirnov and Warnow, 2021b)]. RNASim-500bp has half of its sequences fragmented to an average length of 500 bp (Nguyen *et al.*, 2015), and when we later vary the backbone and query set size, we directly assign full-length sequences to the backbone and the fragments to the query.)

**Table 1.** Dataset statistics (average or range)

Dataset	No. of seqs.	Seq. length	Align length	p-dist.^d^ (avg)
Simulated nucleotide				
1000M1-HF^a^	1000	631.3	3960	0.694
1000M2-HF	1000	634.3	3972	0.683
1000M3-HF	1000	629.6	2723	0.660
1000M4-HF	1000	629.6	2571	0.495
RNASim-500bp^b^	1500–7000	1025.4	21 946	0.408
Biological nucleotide				
CRW	5507–27 643	105.6–1557.2	414–11 856	0.210–0.425
Biological protein				
10AA	303–807	432.7	1745.3	0.671
Homfam^c^	14 950–93 681	149.8	273.5	0.690

*Note*: See Supplementary Tables S4–S7 for additional details.

a The 1000M series data have 20 replicates. 1000M1 has an outlier replicate as indicated in Smirnov and Warnow (2021a), hence removed.

b RNASim-500bp has five replicates (on 5000 full-length sequences and 5000 fragmented sequences) and we sampled different configurations of backbone-query size to benchmark the algorithm.

c HomFam datasets only have reference alignments on a small subset of the sequences. The last two of this row are derived from these small references.

d Average p-dist (p-distance) is the proportion of homologous pairs of letters that are different, where a ‘homologous pair of letters’ is two letters in the same column in the alignment.

We performed two experiments. In Experiment 1, we compare WITCH, WITCH-NG and UPP on both simulated and biological nucleotide datasets [from the Comparative RNA Website (CRW) (Cannone *et al.*, 2002)] where we have reference alignments on the entire set of sequences. In Experiment 2, we compare WITCH, WITCH-NG and UPP on the 10AA dataset (a selected set of 10 protein alignment datasets that have curated alignments) (Gloor *et al.*, 2005; Nguyen *et al.*, 2015; Thompson *et al.*, 2011) and also on the 10 largest HomFam datasets (Sievers *et al.*, 2011) (up to 93 681 sequences), which only have reference alignments on very small subsets of the input sequences.

### 4.1 Evaluation criteria

To evaluate estimated alignments, we use the error metrics computed by FastSP (Mirarab and Warnow, 2011). Briefly, each alignment encodes a set of homology pairs (defined by the columns of the alignment). SPFN (Sum-of-Pairs False Negative rate) is the proportion of true homology pairs (those from the reference alignment) that are not present in the estimated alignment. SPFP (Sum-of-Pairs False Positive rate) is the proportion of estimated homology pairs not found in the reference. Both rates are defined from 0 to 1. For convenience, we often report the average of SPFN and SPFP, sometimes referred as the alignment error or average error. We evaluate the rates across the entire alignment (not just restricted on the query sequences) both to show the final accuracy of the methods as *de novo* alignment methods aligning sequences from scratch and to also take into account the homologies between query sequences and the backbone sequences (which will be ignored if only restricted to the query sequences).

For speed, we record the wall-clock running time of the methods assuming the backbone tree and backbone alignment are given (i.e. ignoring the time used in Phase 0). Aside from the benefit of excluding a shared identical stage for all the methods, this wall-clock running time is exactly the running time for downstream analyses, such as phylogenetic placement or taxon identification, where the backbone alignment and tree are assumed to be already computed.

### 4.2 Computing environment

All experiments were conducted on the Illinois Computing Cluster, a heterogeneous computing cluster where most runs are constrained to four hours with at least 64GB of available memory. For the most time-consuming dataset (16S.B.ALL), we allowed methods to run for more than four hours. We ran all methods across 16 cores.

### 4.3 Other MSA methods

We compare WITCH-NG to WITCH and UPP, each run in default mode, but all three methods used the same backbone alignment and eHMM. See Supplementary Material Section S2 for commands and version numbers.

Methods other than UPP and WITCH were not selected for comparison as prior literature and preliminary results suggest that UPP is more accurate than other published methods (that we know of). For example, prior studies suggest that UPP is more accurate than hmmbuild+hmmalign (Nguyen *et al.*, 2015) and MAFFT—add (Shen *et al.*, 2022a) (among those—add options that can scale to large datasets).

## 5 Results

### 5.1 Experiment 1: Simulated and biological nucleotide datasets

#### 5.1.1 Alignment error

WITCH and WITCH-NG are essentially tied for average alignment accuracy (i.e. average of SPFN and SPFP) and both are more accurate than UPP (Table 2), with the advantage over UPP most noticeable under higher levels of evolution (1000M2-HF and 1000M1-HF). Results on RNASim are shown in the Supplementary Table S1, as the methods have low SPFN (0.092 to 0.102) and SPFP (0.086 to 0.097) error, never differing in error for any model condition by more than 0.004, but when there are differences then UPP is less accurate. When separating into SPFN and SPFP, we see that WITCH and WITCH-NG have higher SPFP than UPP and lower SPFN rates, but the decrease in SPFN is larger than the increase in SPFP, so that the average alignment error is lower for WITCH and WITCH-NG than for UPP.

**Table 2.** Alignment error rates on nucleotide datasets (four simulated and four biological datasets) placing into the same backbone alignment and tree

Dataset	Method	SPFN	SPFP	Avg. error
1000M4-HF	WITCH	**0.014**	**0.010**	**0.012**
	WITCH-NG	**0.014**	**0.010**	**0.012**
	UPP	0.016	**0.010**	0.013
1000M3-HF	WITCH	**0.048**	0.039	**0.043**
	WITCH-NG	**0.048**	0.039	**0.043**
	UPP	0.054	**0.038**	0.046
1000M2-HF	WITCH	**0.128**	**0.106**	**0.117**
	WITCH-NG	**0.128**	**0.106**	**0.117**
	UPP	0.137	**0.106**	0.121
1000M1-HF	WITCH	0.172	0.143	**0.157**
	WITCH-NG	**0.171**	0.143	**0.157**
	UPP	0.182	**0.142**	0.162
5S.3	WITCH	**0.089**	0.086	**0.088**
	WITCH-NG	**0.089**	0.086	**0.088**
	UPP	0.093	**0.085**	0.089
5S.T	WITCH	**0.117**	0.104	**0.110**
	WITCH-NG	**0.117**	0.104	0.111
	UPP	0.120	**0.103**	0.112
16S.3	WITCH	**0.089**	**0.166**	**0.128**
	WITCH-NG	**0.089**	**0.166**	**0.128**
	UPP	0.090	**0.166**	**0.128**
16S.T	WITCH	**0.172**	0.177	**0.175**
	WITCH-NG	**0.172**	0.178	**0.175**
	UPP	0.189	**0.171**	0.180
16S.B.ALL	WITCH	**0.044**	**0.045**	**0.044**
	WITCH-NG	**0.044**	**0.045**	**0.044**
	UPP	0.045	**0.045**	0.045

*Note*: SPFN and SPFP are alignment error rates (lower is better, see text) and ‘Avg. Error’ is the average of the two values, best values boldfaced.

#### 5.1.2 Running time

**Fig. 4.**
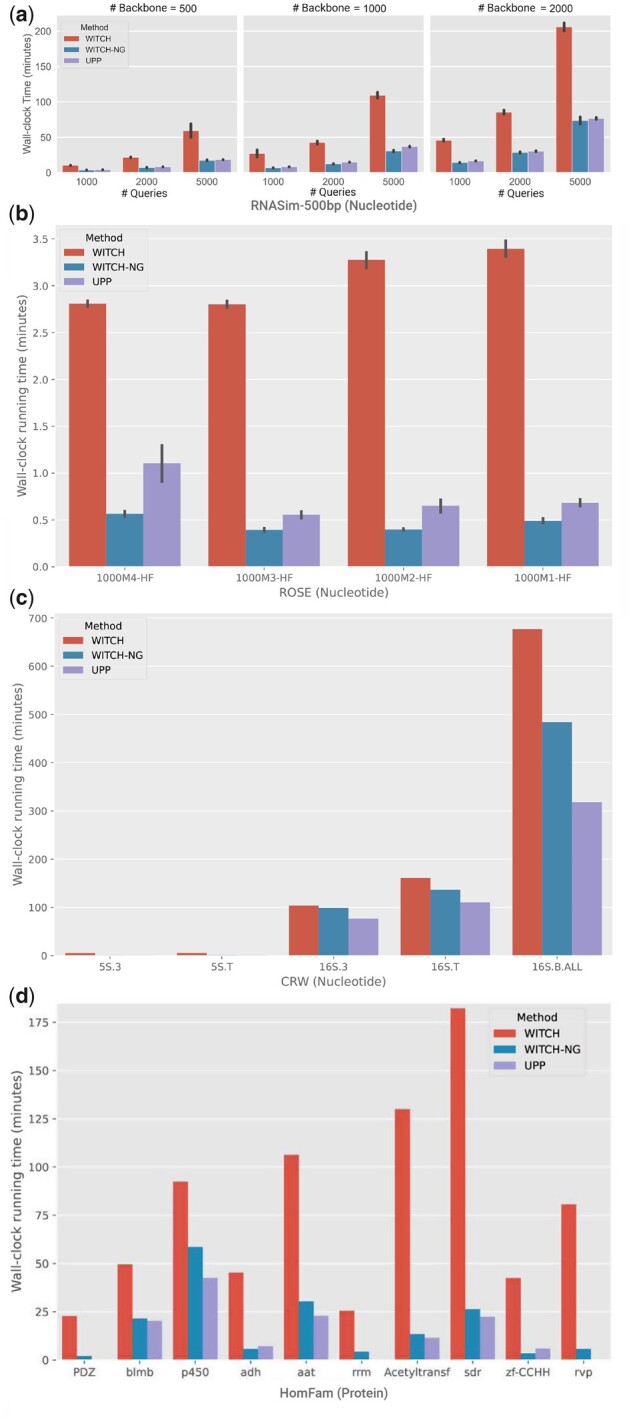
Wall-clock running time (minutes) for Experiments 1 and 2, time measured for Phases 1–4 (i.e. not counting Phase 0). (**a**) Runtimes on RNASim-500bp. WITCH fails on one replicate for the condition with 5000 query sequences and 2000 backbone sequences; results shown here are on only the four replicates where all methods complete. (**b**) Runtimes on ROSE (simulated). (**c**) Runtimes for the CRW datasets. Left-to-right, the number of sequences in each dataset is 5507, 5751, 6323, 7350 and 27 643. (**d**) Runtimes on Homfam datasets. Left-to-right, the number of sequences in each dataset is 14 950, 17 200, 21 013, 21 331, 25 100, 27 610, 46 285, 50 157, 88 345 and 93 681. *Notes*: RNASim and ROSE are HF conditions, so the backbone alignments are on all the full-length sequences. CRW and HomFam analyses have 1000 backbone sequences and the rest are included in the query set. See text for discussion about 10AA datasets (where all methods took <2 min outside Phase 0)

Figure 4 compares runtimes for WITCH, WITCH-NG and UPP on datasets from Experiment 1. WITCH-NG is faster than WITCH across all settings, in some cases much faster (e.g. many of the HomFam datasets). While both methods finished very quickly on some datasets (1000M-HF, 5S.3 and 5S.T), even on those datasets, WITCH-NG was faster than WITCH (Supplementary Table S8). WITCH failed on one replicate of the RNASim datasets (with a backbone of 2000 sequences and 5000 query sequences) due to exceeding the four-hour tine limit, but UPP and WITCH-NG completed on all replicates.

The comparison between WITCH-NG and UPP depends on the dataset, with small differences on the RNASim datasets (often favoring WITCH-NG), bigger differences on the ROSE datasets (favoring WITCH-NG) and three largest CRW datasets (favoring UPP) and variable differences on the HomFam datasets (sometimes favoring UPP and sometimes favoring WITCH-NG). The two methods were essentially tied for speed on the 1000M-HF, 5S.3 and 5S.T datasets (Supplementary Table S8).

**Fig. 5.**
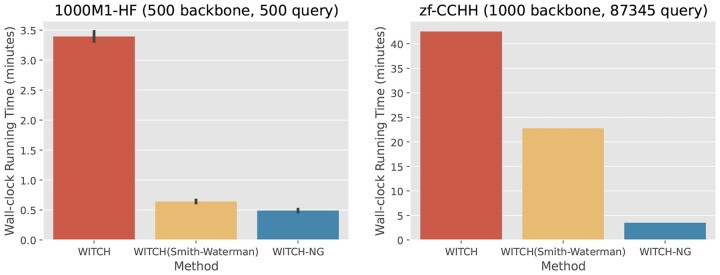
Exploring the effect of the algorithmic change (Smith–Waterman in lieu of default GCM) versus the implementational changes, showing wall-clock running time (minutes, only measured on Phases 1–4) between methods on two very different data. ‘WITCH(Smith–Waterman)’ denotes WITCH but with the GCM step replaced by the described Smith–Waterman step. On 1000M1-HF, the algorithmic change dominates the reduction in running time. On zf-CCHH, the implementation change is as important as the algorithmic change for the running time. On 1000M1-HF, we show averages across replicates with standard error bars

WITCH-NG differs from WITCH in two ways: algorithmic design and algorithmic engineering (implementation). To isolate the impact of the two types of changes, we created a version of WITCH where we replaced WITCH’s GCM subroutine by our described variant of Smith–Waterman. This version of WITCH thus only includes the changes in algorithmic design. We then compare the runtimes of WITCH (the original implementation), ‘WITCH(Smith–Waterman)’ (the modified WITCH just described), and WITCH-NG on two very different datasets used in this study, with the results shown in Figure 5. The simplification in algorithm design is accountable for most (in the case of 1000M1-HF) and roughly half (for zf-CCHH) of the speed up achieved by WITCH-NG. This shows that both the algorithmic design and engineering contribute to the speed up.

WITCH-NG is faster than WITCH in all the conditions we explored, sometimes by a large amount (e.g. the simulated datasets and the 5S datasets from the CRW collection). Thus, WITCH-NG speeds up WITCH on all datasets, sometimes by a great margin. In addition, WITCH-NG is reasonably close to UPP in runtime, while this is not true for WITCH.

### 5.2 Experiment 2: Protein datasets

#### 5.2.1 Alignment accuracy

This experiment focused on two benchmark collections (10AA and Homfam) of protein datasets with reference alignments; here we discuss both runtime and average alignment error for these benchmark collections, with results on each dataset shown in the Supplementary Tables S2 and S3.

On the 10AA datasets (first row in Table 3), WITCH and WITCH-NG have identical average alignment SPFN and SPFP error. UPP has slightly higher SPFN (by 0.003) and slightly lower SPFP (by 0.004) compared to WITCH and WITCH-NG, so that UPP is 0.001 lower in average alignment error than WITCH and WITCH-NG. On the 10AA dataset, WITCH and WITCH-NG achieved essentially identical accuracy, differing more than 0.001 in SPFN or SPFP on only one dataset (Supplementary Table S3).

**Table 3.** Alignment error rates on 10AA and Homfam (averaged across the 10 datasets in each collection)

Dataset	Method	SPFN	SPFP	Avg. error
10AA	WITCH	**0.233**	0.189	0.211
	WITCH-NG	**0.233**	0.189	0.211
	UPP	0.236	**0.185**	**0.210**
Homfam	WITCH	**0.327**	0.124	**0.225**
	WITCH-NG	**0.327**	0.125	0.226
	UPP	0.336	**0.122**	0.229

Boldfaced values are the best results found for each criterion on each dataset.

On the 10 largest HomFam datasets (second row, Table 3), WITCH and WITCH-NG are identical in SPFN but with WITCH better (by 0.001) than WITCH-NG for SPFP; thus, the differences are very small. Compared to WITCH, UPP shows a large increase in SPFN (by 0.009) and a small improvement in SPFP (by 0.02), so that on average UPP is less accurate than both WITCH and WITCH-NG.

#### 5.2.2 Running time

For running times on HomFam (Fig. 4, middle subfigure), we see a dramatic reduction of the running time from WITCH to WITCH-NG in many cases. For example, on 7 of 10 HomFam datasets tested, WITCH-NG achieved at least a 5-fold speedup. Furthermore, WITCH-NG uses nearly the same time as UPP for the HomFam datasets, in most cases only paying a small penalty in running time compared to UPP. On the 10AA datasets, all three methods finished under 2 min for Phases 1–4, with WITCH-NG always faster than WITCH, and faster than UPP on 8 of the 10 datasets (Supplementary Table S3).

## 6 Discussion

WITCH-NG is designed to be a fast version of WITCH. Specifically, WITCH-NG replaces the critical step in WITCH where a collection of extended alignments for the same query sequence is merged into a single extended alignment for that query sequence. This consensus alignment step is performed in WITCH through a computationally intensive heuristic, the Graph Clustering Merger from Smirnov and Warnow (2021a), which uses Markov Clustering (MCL) followed by an A* heuristic. In contrast, WITCH-NG obtains its consensus clustering using a local alignment step computed using Smith–Waterman. Since the GCM method and the local alignment method essentially aim to produce the same consensus (i.e. optimizing the Maximum Weight Trace for Alignment Merging), WITCH-NG is designed to produce an alignment similar to that of WITCH, but to do it faster.

Our study shows that WITCH-NG and WITCH produced nearly identical alignments, never differing in more than 0.001 SPFN or SPFP on any biological dataset (Supplementary Table S9). This very high similarity is not surprising, given that both WITCH and WITCH-NG optimize the same criterion (MWT-AM) when computing the consensus alignment.

We also saw a substantial speed up in WITCH-NG over WITCH in many datasets. This is also to be expected, since GCM is heuristic and both its steps are computationally intensive while the Smith–Waterman method is deterministic and polynomial time. We also saw that while the improvement in runtime varied, the improvement in runtime could be very large. Finally, we note that the runtime improvement resulted from both the algorithmic innovation (using Smith–Waterman for the consensus alignment instead of using the Graph Clustering Merger) as well as from the improved algorithm engineering in WITCH-NG.

## 7 Conclusion

MSA is a step in many bioinformatics pipelines, and yet datasets that are large, highly divergent or contain fragmentary sequences are difficult to align with high accuracy. WITCH is a MSA method that aims to address the challenge of aligning datasets with many fragmentary sequences, and WITCH-NG is modification of the WITCH alignment method that aims to improve the running time without hurting accuracy. Our study shows that WITCH and WITCH-NG methods produce nearly identical alignments, but that WITCH-NG is generally faster, and sometimes greatly faster. The high similarity between WITCH and WITCH-NG alignments explains why WITCH-NG provides high accuracy in estimated alignments and suggests that WITCH-NG should be useful for those applications where WITCH alignments have already been shown to be beneficial, such as phylogeny estimation (Shen *et al.*, 2022b).

This study suggests several directions for future work. For example, in WITCH-NG, we used a naive implementation of the naive Smith–Waterman algorithm to add a query sequence to the backbone alignment. The speed up we obtain is therefore likely to be improved, given the many optimized variants of DP sequence alignment algorithms that are much faster on modern architectures [e.g. ‘striped Smith–Waterman’ (Farrar, 2007)].

It is also worth noting that WITCH-NG addresses only one of the aspects of the WITCH algorithm design that leads to a long runtime: how it combines extended alignments for each of the query sequence into a consensus extended alignment, so that the query sequence can be added to the backbone alignment. However, WITCH has another computationally intensive step not addressed in WITCH-NG, where it compares each query sequence to each HMM in the ensemble of HMMs computed by WITCH for the backbone alignment. Modifying this design so that WITCH-NG only does a few of the query-HMM comparisons could potentially further speed up WITCH-NG and should be evaluated. One potential such modification could use the approach in UPP2 (Park *et al.*, 2023) to reduce the number of query-HMM comparisons used in UPP, and which enabled UPP2 to match the accuracy of UPP but be faster for large datasets.

Other future research could evaluate the impact of WITCH-NG for use in bioinformatics pipelines that use MSAs (e.g. phylogeny estimation, as mentioned above). WITCH-NG can also be used directly to add sequences into alignments, a problem that arises in updating existing alignments and trees as new sequences are assembled and in microbiome analysis. WITCH-NG should be evaluated for use in these applications, especially for those analyses where runtime is extremely important (e.g. taxon identification in metagenomics).

## Supplementary Material

vbad024_Supplementary_Data
