# Supplementary material for: WITCH-NG: efficient and accurate alignment of datasets with sequence length heterogeneity
Source: Bioinform Adv. 2023 Mar 6;3(1):vbad024. doi: 10.1093/bioadv/vbad024 (PMC10035637; doi:10.1093/bioadv/vbad024)
Supplement: vbad024_Supplementary_Data [file vbad024_supplementary_data.pdf]

Supplementary Materials for "WITCH-NG"

Baqiao Liu and Tandy Warnow

March 1, 2023

Contents

1 Additional Tables **2**

2 Software Versions and Commands **11**

2.1 Methods for Adding Query Sequences to Alignments 11
2.2 HMMER Commands 11
2.3 Other Commands 11

3 Dataset Availability **12**

List of Tables

S1 Alignment error on RNA-Sim 2
S2 Alignment error on 10 HomFam datasets 3
S3 Alignment error and running times on the 10AA datasets. 4
S4 Expanded empirical properties for simulated datasets 5
S5 Expanded dataset properties for the 10AA dataset 6
S6 Expanded dataset properties of the individual (full) HomFam datasets 7
S7 Expanded empirical properties for CRW datasets 8
S8 Running times for 1000M-HF, 5S.3, and 5S.T nucleotide datasets 9
S9 Comparison of WITCH and WITCH-NG alignments on the biological datasets 10

1 Additional Tables

Backbone Size	Query Size	Method	SPFN	SPFP	Avg. Error
500	1000	WITCH	**0.100**	**0.097**	**0.099**
		WITCH-NG	**0.100**	**0.097**	**0.099**
		UPP	0.102	**0.097**	**0.099**
	2000	WITCH	**0.099**	**0.094**	**0.097**
		WITCH-NG	**0.099**	**0.095**	**0.097**
		UPP	0.102	**0.094**	**0.098**
	5000	WITCH	**0.099**	**0.093**	**0.096**
		WITCH-NG	**0.098**	**0.093**	**0.096**
		UPP	0.102	**0.093**	0.097
1000	1000	WITCH	**0.096**	**0.093**	**0.095**
		WITCH-NG	**0.096**	**0.093**	**0.095**
		UPP	**0.097**	**0.093**	**0.095**
	2000	WITCH	**0.096**	**0.091**	**0.094**
		WITCH-NG	**0.096**	**0.092**	**0.094**
		UPP	0.098	0.091	**0.094**
	5000	WITCH	**0.096**	**0.090**	**0.093**
		WITCH-NG	**0.095**	**0.090**	**0.093**
		UPP	0.098	**0.090**	**0.094**
2000	1000	WITCH	**0.093**	**0.088**	**0.091**
		WITCH-NG	**0.093**	**0.088**	**0.091**
		UPP	**0.094**	**0.088**	**0.091**
	2000	WITCH	**0.093**	**0.088**	**0.090**
		WITCH-NG	**0.093**	**0.088**	**0.090**
		UPP	**0.094**	**0.087**	**0.091**
	5000	WITCH-NG	**0.092**	**0.086**	**0.089**
		WITCH	**0.092**	**0.086**	**0.089**
		UPP	0.094	**0.086**	0.090

Table S1: Alignment error rates on RNASim-500bp, averaged over five replicates. WITCH timed out on 1/5 replicates on the 200 backbone 5000 query configuration, hence for that configuration all methods are averaged across four replicates. SPFN (sum-of-pairs false negative) refers to the proportion of pairwise homologies in the reference alignment missing in the estimated alignment, SPFP (sum-of-pairs false positive) refers to the proportion of homologies in the estimated alignment that are not in the reference alignment, and "Avg. Error" is the average of these two values. Best values (with ties within 0.001) are boldfaced.

Dataset	Method	SPFN	SPFP	Avg. Error
PDZ	WITCH	**0.211**	**0.096**	**0.154**
	WITCH-NG	**0.211**	**0.096**	**0.154**
	UPP	0.239	0.107	0.173
blmb	WITCH	**0.300**	0.139	0.220
	WITCH-NG	**0.300**	0.142	0.221
	UPP	0.310	**0.122**	**0.216**
p450	WITCH	0.279	0.183	**0.231**
	WITCH-NG	**0.277**	0.186	**0.231**
	UPP	0.298	**0.175**	0.237
adh	WITCH	**0.638**	**0.003**	**0.321**
	WITCH-NG	**0.638**	**0.003**	**0.321**
	UPP	0.641	**0.003**	**0.322**
aat	WITCH	**0.182**	**0.106**	**0.144**
	WITCH-NG	**0.182**	**0.106**	**0.144**
	UPP	**0.183**	**0.106**	**0.145**
rrm	WITCH	**0.246**	**0.134**	**0.190**
	WITCH-NG	**0.247**	0.136	**0.191**
	UPP	0.253	**0.134**	0.194
Acetyltransf	WITCH	**0.548**	0.167	0.358
	WITCH-NG	**0.548**	0.157	0.352
	UPP	0.550	**0.146**	**0.348**
sdr	WITCH	**0.404**	**0.140**	**0.272**
	WITCH-NG	**0.405**	0.148	0.276
	UPP	0.421	0.147	0.284
zf-CCHH	WITCH	**0.186**	**0.055**	**0.120**
	WITCH-NG	**0.186**	**0.055**	**0.120**
	UPP	**0.186**	**0.056**	**0.121**
rvp	WITCH	**0.273**	**0.218**	**0.246**
	WITCH-NG	**0.273**	**0.218**	**0.246**
	UPP	0.281	0.227	0.254
Average across ten	WITCH	**0.327**	0.124	**0.225**
	WITCH-NG	**0.327**	0.125	**0.226**
	UPP	0.336	**0.122**	0.229

Table S2: Alignment error rates on the ten largest HomFam datasets. SPFN (sum-of-pairs false negative) refers to the proportion of pairwise homologies in the reference alignment missing in the estimated alignment, SPFP (sum-of-pairs false positive) refers to the proportion of homologies in the estimated alignment that are not in the reference alignment, and "Avg. Error" is the average of these two values. Best values (with ties within 0.001) are boldfaced.

Dataset	Method	Time (s)	SPFN	SPFP	Avg. Error
1GADBL_100	UPP	7.22	**0.033**	**0.034**	**0.034**
	WITCH	9.75	**0.033**	**0.034**	**0.034**
	WITCH-NG	**4.09**	**0.033**	**0.034**	**0.034**
coli_epi_100	UPP	2.95	**0.036**	**0.039**	**0.038**
	WITCH	4.73	**0.036**	**0.039**	**0.038**
	WITCH-NG	**1.29**	**0.036**	**0.039**	**0.038**
RV100_BBA0039	UPP	20.42	**0.071**	0.094	0.083
	WITCH	37.75	0.075	**0.086**	**0.080**
	WITCH-NG	**11.6**	0.075	**0.086**	**0.080**
RV100_BBA0067	UPP	15.37	**0.220**	**0.276**	**0.248**
	WITCH	40.46	**0.226**	0.274	0.250
	WITCH-NG	**12.27**	**0.226**	0.273	0.250
RV100_BBA0081	UPP	**20.59**	**0.494**	0.683	**0.589**
	WITCH	46.71	0.499	**0.681**	0.590
	WITCH-NG	26.36	0.499	**0.681**	0.590
RV100_BBA0101	UPP	21.49	**0.251**	0.324	**0.287**
	WITCH	70.77	0.257	**0.322**	0.290
	WITCH-NG	**20.87**	0.257	**0.322**	0.290
RV100_BBA0117	UPP	4.36	**0.147**	0.155	**0.151**
	WITCH	13.64	0.150	**0.151**	**0.151**
	WITCH-NG	**1.15**	**0.151**	0.151	**0.151**
RV100_BBA0134	UPP	**36.46**	**0.167**	0.253	**0.210**
	WITCH	108.54	0.171	**0.253**	0.212
	WITCH-NG	48.96	0.178	**0.255**	0.216
RV100_BBA0154	UPP	8.09	**0.201**	**0.233**	**0.217**
	WITCH	10.23	0.202	**0.233**	**0.217**
	WITCH-NG	**4.37**	0.202	**0.233**	**0.217**
RV100_BBA0190	UPP	22.06	**0.219**	**0.249**	**0.234**
	WITCH	34.37	**0.219**	**0.249**	**0.234**
	WITCH-NG	**16.73**	**0.219**	**0.249**	**0.234**

Table S3: Alignment error rates and running times on the 10AA datasets. Runtime is restricted to Phases $1-4$, and so does not include the time to compute the backbone alignment and tree (which is common to all three methods).

**Name**	**Type**	**# seqs.**	**Seq. length**	**Align length**	**% gappy**	**p-dist.**	
						**avg.**	**max**
1000M1-HF	Sim NT	1000	631.3	3960	84.0	0.694	1.0
1000M2-HF	Sim NT	1000	634.3	3972	83.8	0.683	1.0
1000M3-HF	Sim NT	1000	629.6	2723	76.7	0.660	1.0
1000M4-HF	Sim NT	1000	629.6	2571	75.3	0.495	1.0
RNASim-LF	Sim NT	1500-7000	1025.44	21946	95.3	0.408	1.0

Table S4: Expanded dataset statistics for simulated datasets.

Name	Type	Align length	# seqs.	% gappy	Avg. Seq Length	Avg. p-dist	Max p-dist
1GADBL_100	Bio AA	490	561	33.7%	324.9	0.4573	0.715
coli_epi_100	Bio AA	150	320	11.3%	133.1	0.5829	0.871
RV100_BBA0039	Bio AA	2696	807	85.3%	395.1	0.4153	1.000
RV100_BBA0067	Bio AA	1092	410	57.5%	463.7	0.7827	0.922
RV100_BBA0081	Bio AA	1693	353	65.4%	585.8	0.8631	1.000
RV100_BBA0101	Bio AA	4214	509	88.3%	492.3	0.7844	1.000
RV100_BBA0117	Bio AA	110	460	48.4%	56.7	0.7537	1.000
RV100_BBA0134	Bio AA	3186	717	85.2%	470.2	0.7286	1.000
RV100_BBA0154	Bio AA	1275	303	59.3%	518.5	0.6598	0.850
RV100_BBA0190	Bio AA	2547	397	65.2%	886.3	0.6884	1.000

Table S5: Expanded dataset properties for the 10AA datasets, based on the reference alignments for these datasets.

Name	Type	Align length	# seqs.	% gappy	Avg. Seq Length	Avg. p-dist	Max p-dist
PDZ	Bio AA	3730	14944	97.8%	80.9	0.7552	0.970
blmb	Bio AA	13246	17194	98.5%	192.4	0.8008	1.000
p450	Bio AA	6848	21001	95.2%	331.5	0.7519	1.000
adh	Bio AA	5761	21326	97.9%	123.6	0.7792	1.000
aat	Bio AA	12667	25090	97.3%	337.8	0.8090	1.000
rrm	Bio AA	3138	27590	97.9%	67.4	0.7604	1.000
Acetyltransf	Bio AA	16044	46279	99.5%	83.0	0.8032	1.000
sdr	Bio AA	14468	50144	98.9%	163.2	0.7444	1.000
zf-CCHH	Bio AA	2287	88330	99.0%	23.3	0.6332	0.941
rvp	Bio AA	569	93675	83.4%	94.3	0.1275	1.000

Table S6: Dataset properties of the individual HomFam datasets. Due to reference sequences present only on a small subset of the sequences, the dataset properties are measured on MAGUS alignments on the unaligned sequences.

**Name**	**Type**	**# seqs.**	**Seq. length**	**Align length**	**% gappy**	**p-dist.**	
						**avg.**	**max**
5S.3	Bio NT	5507	105.6	414	74.5	0.418	1.0
5S.T	Bio NT	5751	106.2	436	75.6	0.425	1.0
16S.3	Bio NT	6323	1557.2	8716	82.1	0.315	0.833
16S.T	Bio NT	7350	1492.1	11856	87.4	0.345	0.901
16S.B.ALL	Bio NT	27643	1371.9	6857	80.0	0.210	0.769

Table S7: Expanded dataset statistics for CRW datasets, based on the reference alignments from [1].

Dataset	Method	**Time (min)**
1000M1	WITCH	3.4
	WITCH-NG	**0.5**
	UPP	0.7
1000M2	WITCH	3.3
	WITCH-NG	**0.4**
	UPP	0.7
1000M3	WITCH	2.8
	WITCH-NG	**0.4**
	UPP	0.6
1000M4	WITCH	2.8
	WITCH-NG	**0.6**
	UPP	1.1
5S.3	WITCH	5.8
	WITCH-NG	1.2
	UPP	**1.1**
5S.T	WITCH	5.9
	WITCH-NG	1.4
	UPP	**1.3**

Table S8: Running times for six nucleotide datasets (4 simulated and 2 biological) that had short (less than ten minutes) in running time for Phases 1–4.

**Dataset**	**SPFN**	**SPFP**	**Avg. Error**
CRW (5)	0.000	0.000	0.000
10AA (10)	0.000	0.000	0.000
HomFam (10)	0.000	0.001	0.001

Table S9: Average SPFN and SPFP rates comparing WITCH and WITCH-NG alignments on the biological datasets, treating WITCH as the reference alignments with WITCH-NG as the estimated alignments.

2 Software Versions and Commands

2.1 Methods for Adding Query Sequences to Alignments

These methods assume the existence of the following three files:

- `$bb_aln`, (path to) the backbone alignment
- `$bb_tre`, the backbone tree
- `$q`, the query sequences

In addition, WITCH and UPP require specifying the type of "molecule" in the input, either "`dna`", "`rna`", or "`amino`". Let `$molecule` be this variable. Both WITCH and UPP require a working directory, which we denote by `$work_dir`. The number 16 that occurs across the commands is the number of threads we used.

We ran UPP version 4.5.2, with the following command:

```
python3 run_upp.py -m $molecule -x 16 -s $q -a $bb_aln -t $bb_tre -d $work_dir -o
    $output_suffix
```

We ran WITCH version 0.2.1, with the following command:

```
python3 witch.py --molecule $molecule -t 16 -q $q -b $bb_aln -e $bb_tre -d $work_dir
```

We ran WITCH-NG version 0.0.2, with the following command:

```
witch-ng add --threads 16 -i $q -b $bb_aln -t $bb_tre -o $output_path
```

2.2 HMMER Commands

We invoke HMMER (version 3.1b2 to keep it consistent with WITCH) inside WITCH-NG as subprocesses. The commands are given below:

```
hmmbuild --cpu 0 --informat afa --ere 0.59 --symfrac 0.0 $hmm_outpath -
hmmsearch --cpu 0 --noali --max -E 999999999 $hmm_path -
hmmalign --informat fasta --outformat afa $hmm_path -
```

2.3 Other Commands

For computing the alignment error statistics, we used FastSP [5] (v1.7.1) with the following command:

```
java -jar FastSP.jar -ml -e $est -r $ref
```

For the "WITCH(Smith-Waterman)" method, we used a custom version of WITCH, located at [3], with the exact same commands used for WITCH listed above.

For aligning the backbone alignment and to infer trees on the backbone alignment, we either directly ran WITCH (which will as a first step split the dataset and align the backbone alignment, then inferring a tree on the backbone alignment) terminating it once the backbone alignment and tree is produced, or we manually align the backbone sequences and infer trees on the backbone alignment using the same methods (MAGUS and FastTree).

For running WITCH to separate the backbone and queries, and to produce the backbone alignment and tree, we used:

```
python3 witch.py -t 16 -i $unaln_seqs -d $work_dir
```

For running MAGUS (0.1.0b2), we used:

```
python3 magus.py -np 16 -i $unaln_seqs -d $work_dir -o $output_aln_path
```

For running FastTree (v2.1.11 SSE3), we used:

```
FastTreeMP -lg $bb_aln > $bb_tre # for AA data
FastTreeMP -nt -gtr $bb_aln > $bb_tre # for NT data
```

3 Dataset Availability

We list the sources of our datasets here, with links to the download pages.

- 1000M-HF: [11]
- RNASim-500bp, originally generated in [4] and preprocessed by [8]: [6]
- CRW [1]
  - 5S series: [9]
  - 16S series: [7]
- 10AA (based on [12, 2]): [6]
- HomFam: [10]

References

[1] Jamie J. Cannone, Sankar Subramanian, Murray N. Schnare, James R. Collett, Lisa M. D'Souza, Yushi Du, Brian Feng, Nan Lin, Lakshmi V. Madabusi, Kirsten M. Müller, Nupur Pande, Zhidi Shang, Nan Yu, and Robin R. Gutell. The Comparative RNA Web (CRW) Site: an online database of comparative sequence and structure information for ribosomal, intron, and other RNAs. *BMC Bioinformatics*, 3(1):2, January 2002.

[2] Gregory B. Gloor, Louise C. Martin, Lindi M. Wahl, and Stanley D. Dunn. Mutual information in protein multiple sequence alignments reveals two classes of coevolving positions. *Biochemistry*, 44(19):7156–7165, 2005. PMID: 15882054.

[3] Baqiao Liu. GitHub site for WITCH-NG using Smith-Waterman, 2023. Available at `https://github.com/RuneBlaze/WITCH`, last accessed Feb 28, 2023.

[4] Siavash Mirarab, Nam Nguyen, Sheng Guo, Li-San Wang, Junhyong Kim, and Tandy Warnow. PASTA: Ultra-Large Multiple Sequence Alignment for Nucleotide and Amino-Acid Sequences. *Journal of Computational Biology*, 22(5):377–386, May 2015.

[5] Siavash Mirarab and Tandy Warnow. FastSP: linear time calculation of alignment accuracy. *Bioinformatics*, 27(23):3250–3258, 2011.

[6] Nam-phuong Nguyen, Siavash Mirarab, Keerthana Kumar, and Tandy Warnow. Datasets for publication "Ultra-large alignments using phylogeny-aware profiles", June 2015. `https://sites.google.com/eng.ucsd.edu/datasets/alignment/pastaupp`, last accessed Feb 28, 2023.

[7] Nam-phuong Nguyen, Siavash Mirarab, Keerthana Kumar, and Tandy Warnow. Preprocessed 16s and 23s datasets, June 2015. `https://sites.google.com/eng.ucsd.edu/datasets/alignment/16s23s`, last accessed Feb 28, 2023.

[8] Nam-phuong Nguyen, Siavash Mirarab, Keerthana Kumar, and Tandy Warnow. Ultra-large alignments using phylogeny-aware profiles. *Genome Biology*, 16(1):124, June 2015.

[9] Chengze Shen, Paul Zaharias, and Tandy Warnow. Datasets for publication "MAGUS+eHMMs: Improved multiple sequence alignment accuracy for fragmentary sequences", 2021. `https://doi.org/10.13012/B2IDB-2419626_V1`, last accessed, Feb 28, 2023.

[10] Fabian Sievers, Andreas Wilm, David Dineen, Toby J. Gibson, Kevin Karplus, Weizhong Li, Rodrigo Lopez, Hamish McWilliam, Michael Remmert, Johannes Söding, Julie D. Thompson, and Desmond G. Higgins. Homfam datasets, for publication "Fast, scalable generation of high-quality protein multiple sequence alignments using Clustal Omega", October 2011. `http://www.clustal.org/omega/homfam-20110613-25.tar.gz`, last accessed Feb 28, 2023.

[11] Vladimir Smirnov and Tandy Warnow. Datasets for publication "Phylogeny estimation given sequence length heterogeneity", 2020. `https://doi.org/10.5061/dryad.95x69p8h8`, last accessed March 1, 2023.

[12] Julie D. Thompson, Benjamin Linard, Odile Lecompte, and Olivier Poch. A comprehensive benchmark study of multiple sequence alignment methods: Current challenges and future perspectives. *PLOS ONE*, 6(3):1–14, March 2011.
